# Nanomaterial-based biohybrid hydrogel in bioelectronics

**DOI:** 10.1186/s40580-023-00357-7

**Published:** 2023-02-10

**Authors:** Minkyu Shin, Joungpyo Lim, Joohyun An, Jinho Yoon, Jeong-Woo Choi

**Affiliations:** 1 Department of Chemical & Biomolecular Engineering, Sogang University, Seoul, 04170 Republic of Korea; 2 Department of Biomedical-Chemical Engineering, The Catholic University of Korea, Bucheon, 14662 Republic of Korea

**Keywords:** Nanomaterial, Biohybrid hydrogel, Bioelectronics, Biorobotics, Flexible devices

## Abstract

Despite the broadly applicable potential in the bioelectronics, organic/inorganic material-based bioelectronics have some limitations such as hard stiffness and low biocompatibility. To overcome these limitations, hydrogels capable of bridging the interface and connecting biological materials and electronics have been investigated for development of hydrogel bioelectronics. Although hydrogel bioelectronics have shown unique properties including flexibility and biocompatibility, there are still limitations in developing novel hydrogel bioelectronics using only hydrogels such as their low electrical conductivity and structural stability. As an alternative solution to address these issues, studies on the development of biohybrid hydrogels that incorporating nanomaterials into the hydrogels have been conducted for bioelectronic applications. Nanomaterials complement the shortcomings of hydrogels for bioelectronic applications, and provide new functionality in biohybrid hydrogel bioelectronics. In this review, we provide the recent studies on biohybrid hydrogels and their bioelectronic applications. Firstly, representative nanomaterials and hydrogels constituting biohybrid hydrogels are provided, and next, applications of biohybrid hydrogels in bioelectronics categorized in flexible/wearable bioelectronic devices, tissue engineering, and biorobotics are discussed with recent studies. In conclusion, we strongly believe that this review provides the latest knowledge and strategies on hydrogel bioelectronics through the combination of nanomaterials and hydrogels, and direction of future hydrogel bioelectronics.

**Graphical Abstract:**

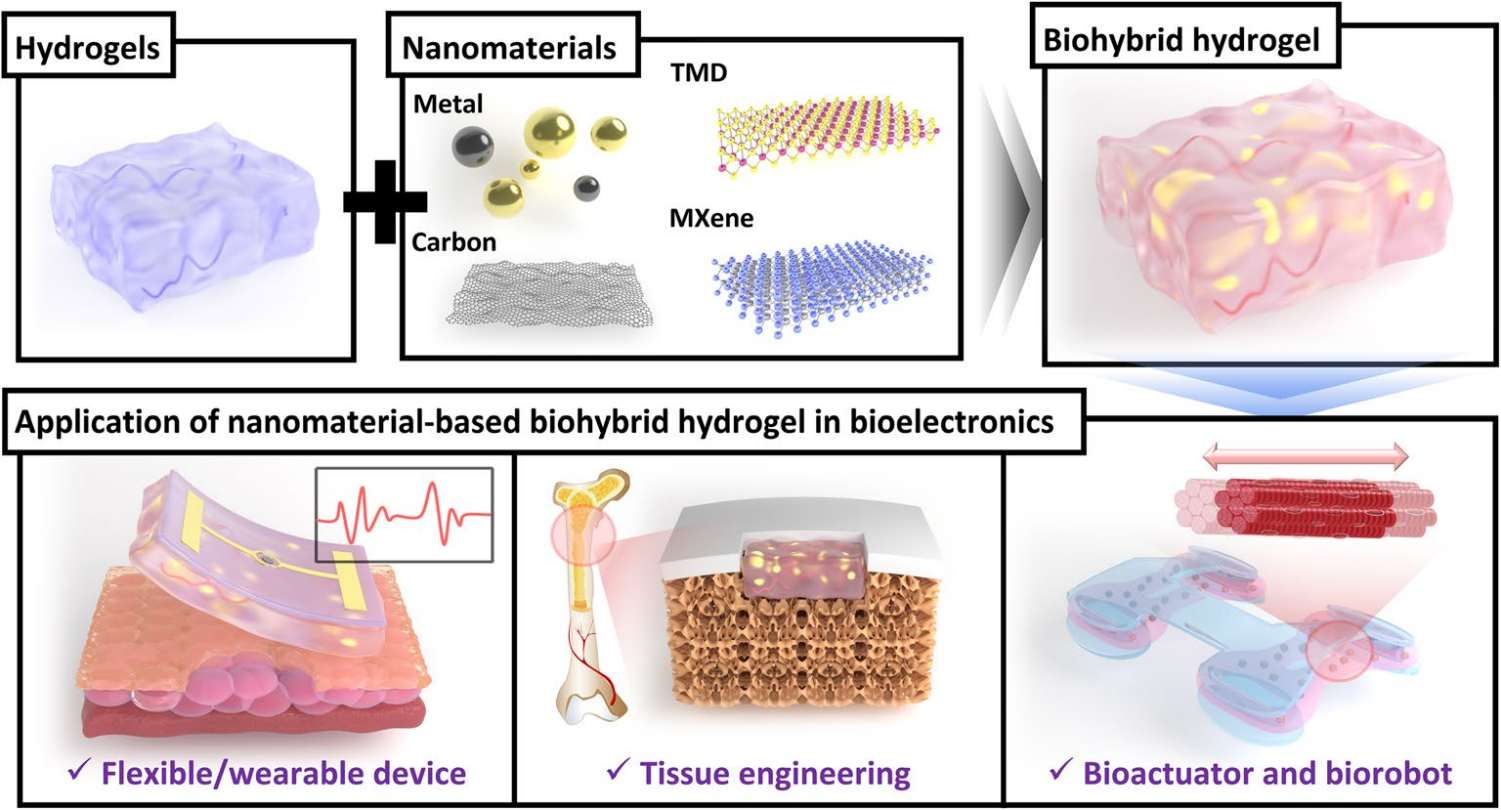

## Introduction

Bioelectronics involve the incorporation of electronics and biological materials, using biomolecule-based inorganic/organic materials for developing electrical/electrochemical biosensors, functional bioelectronic chips or actuators [1, 2]. Up to now, numerous researches on bioelectronics have been conduted by using organic or inorganic materials. However, since organic/inorganic materials-based bioelectronic devices have the rigid physical properties and low biocompatibility, it is difficult to use them for practical researches such as tissue regeneration and other biomedical applications [3, 4]. As a solution to address these limitations of organic/inorganic materials-based bioelectronic devices, hydrogels are being investigated a lot. The hydrogels are three-dimensional (3D) crosslinked-hydrophilic network polymers that can absorb a large amount of water, forming a well-defined structure [5, 6]. Owing to their superior properties including flexibility and high swelling ability, hydrogels have been applied in various research fields including microfluidics and soft robotics. In particular, compared with polymers, hydrogels exhibit high biocompatibility due to their properties including high moisture content, biodegradability, and porous structure, so they can be used for developing biosensors, drug delivery systems, engineered tissue, and other biological applications [7, 8]. Particularly, the hydrogels have shown tremendous potential for the development of bioelectronic devices due to their biological and electrical properties [9–11]. For instance, hydrogels have been used as a component of bioelectronic devices such as electrodes to connect the body to wearable biosensors [12–14]. Hydrogels are effectively attached to the human skin due to their hydrophilicity, softness, and flexibility, and contribute to the highly sensitive biosensing by the electrode. In addition, hydrogels have been implanted directly into tissues for biomedical applications, for instance, to heal a damaged sciatic nerve for biomedical application. Due to their moisture-rich and conductive properties, hydrogels can be fixed in cells and tissues for recovery of the electrophysiological signal processing [15, 16]. In addition to these applicable fields of the hydrogels, in recent years, research on biorobots has received a lot of attention because of their applicability in the biological and biomedical fields, including drug delivery, disease models, and sensing systems [17]. For instance, biorobots can simulate or mimic various functions of living organisms such as the movement and contraction of muscle, blood vessels, and nerve cells. However, for achieving the efficient driving of the biorobots, it is necessary to connect the bioelectronics with tissues or hydrogels to precisely regulate the movement of biorobots by electrical stimulation or by supplying them with electrical energy [18–20]. Moreover, beyond the widely studied soft robots and actuators, which are composed of inorganic materials and polymers, the hydrogels combined with biomaterials could be used to develop novel biorobots that could replace soft robots.

**Fig. 1:**
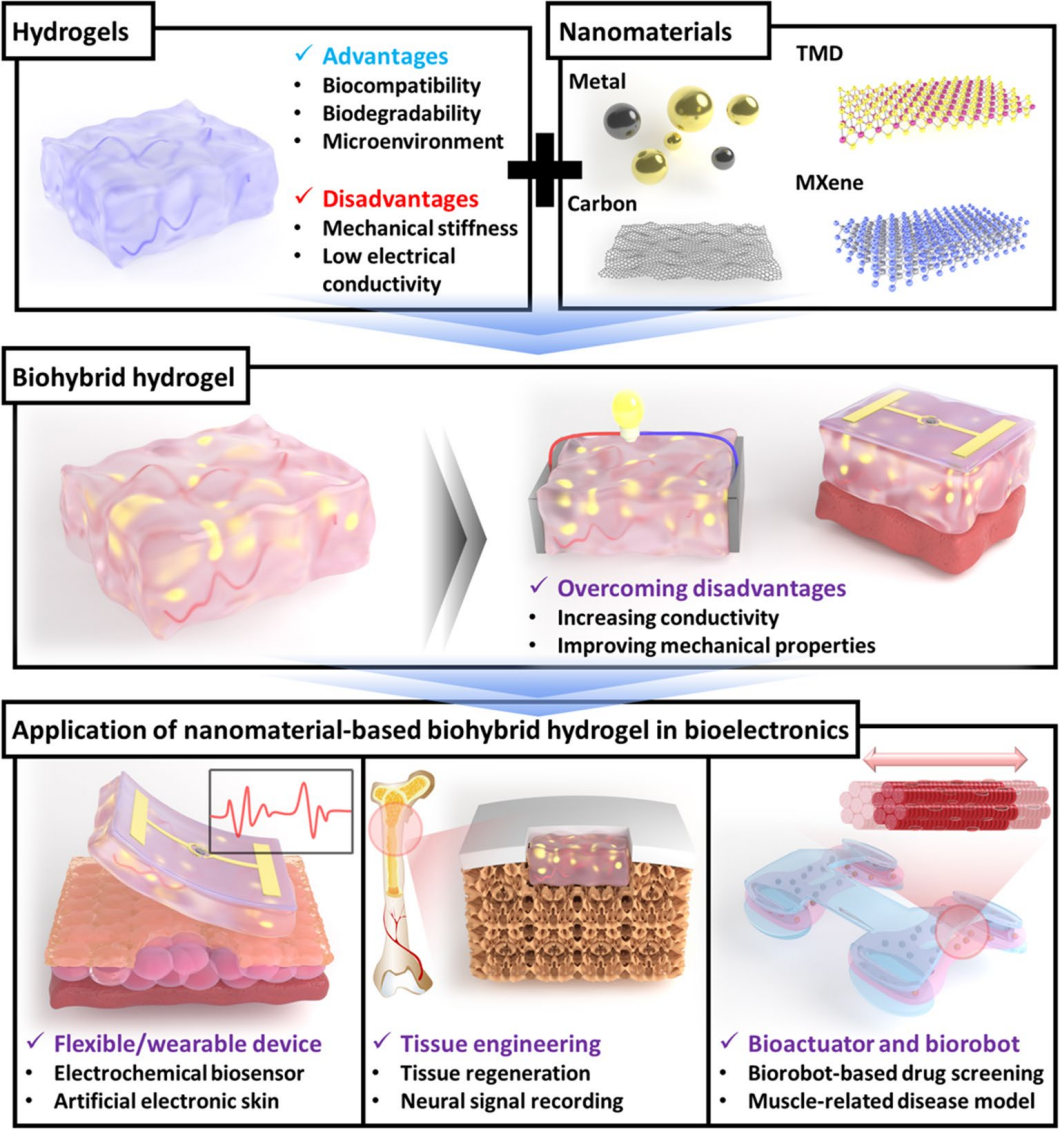
Schematic diagram of nanomaterial-based biohybrid hydrogel in bioelectronics to develop flexible/wearable bioelectronic device, tissue engineering, and biorobot

Although hydrogels are being largely studied for bioelectronics application, they have several limitations, such as relatively low structural stability, conductivity, and low cell adhesion properties. Thus, biohybrid hydrogel developed by combination of nanomaterials and hydrogels can suggest the solution to overcome these limitations of conventional hydrogels through the offer of the synergic effect based on the excellent properties from each component such as the biocompatibility from hydrogel and the conductivity from nanomaterials [21, 22]. For example, nanomaterials show advantages in bioelectronics applications, increasing conductivity and structural stability as well as improving biocompatibility by combining with biomaterials such as peptides, nucleic acids, and enzymes [23, 24]. In addition, nanomaterials can be utilized to address the limitations of hydrogels by combining with them. Thus, biohybrid hydrogels are expected to contribute to the development of novel bioelectronics such as wearable or flexible bioelectronic devices for biomedical and biorobotic applications (Fig. 1) [25].

In this review, biohybrid hydrogel and their applications in bioelectronics are discussed, focusing on recent researches. Firstly, nanomaterials and hydrogels that compose biohybrid hydrogels are presented according to the type of nanomaterials (metal, carbon, transition metal dichalcogenides, and MXene) and hydrogels (natural and biocompatible synthetic). Next, applications of biohybrid hydrogels in bioelectronics are described based on their classification as flexible/wearable bioelectronic devices, bioelectronic devices for tissue engineering, and biorobotic applications. Then, the current limitations and future perspectives of biohybrid hydrogels are discussed and insights into next-generation bioelectronics are presented.

## Nanomaterials and hydrogels for biohybrid hybrogels

### Nanomaterials

Nanomaterials, which generally have sizes in the lower nanometer range, have attracted a lot of attention because of their exceptional mechanical, optical, electrical, and biological properties [26]. Nanomaterials have been applied in many research fields including electronics, medicine, environment, and biotechnology. Hence, numerous studies have reported the combination of nanomaterials with other materials including biomolecules to achieve synergetic effects [27].

**Fig. 2:**
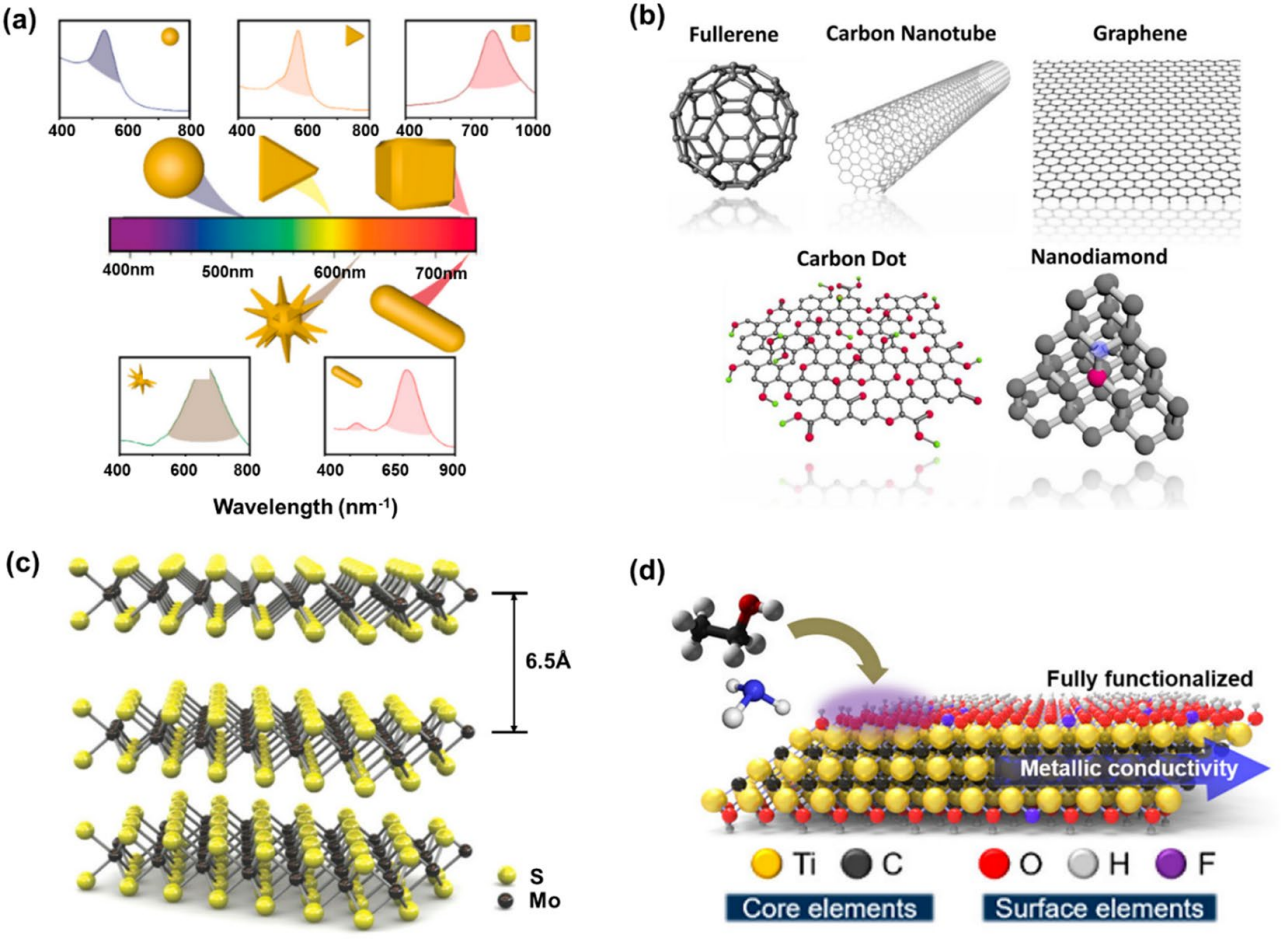
**a** Schematic diagram of the localized surface plasmon resonance (LSPR) shift on Au nanomaterials depending on their different shapes (nanosphere, prism, cube, star, or rod). Reproduced with permission from [30], copyright MDPI, 2020. **b** Schematic images of the structures of representative allotropes of carbon nanomaterials. Reproduced with permission from [34], copyright American Chemical Society, 2015. **c** Schematic illustration of the crystal structure of MoS_2_, which consists of a transitional metal (Mo) atomic layer sandwiched between two chalcogen (S) atomic layers. Reproduced with permission from [37], copyright American Chemical Society, 2015. **d** Schematic illustration of the atomic structure and constituting elements of the Ti_3_C_2_T_x_ MXene. Reproduced with permission from [39], copyright American Chemical Society, 2018

In particular, research on biohybrid hydrogels, aiming to maximize the electrical and biological properties of the hydrogels, has been conducted in recent years [28]. Various types of nanomaterials including metal nanomaterials, carbon nanomaterials, transition metal dichalcogenides (TMDs), and MXene have been combined with hydrogels to improve the structural, optical, physical, and chemical properties of the resulting composites [29]. Metal nanomaterials are the most commonly used for producing biohybrid hydrogels because of their high biocompatibility, optical polarizability, and catalytic activity. The two most often utilized metal nanomaterials are Au and Ag, and depending on their shape and size, they may induce various quantum effects (Fig. 2a) [30]. When light resonates with the free electrons in a nanomaterial, the surface plasmon resonance (SPR) phenomenon is produced, which appears as color variation [31, 32]. Therefore, metal nanomaterials are often employed as sensing components in colorimetric assays. Furthermore, basically, the metal has the superior conductivity compared to the other mateirlas, so the use of metal nanomaterials can provide the excellent conductivity which is eseentially required to develop the bioelectronic funtions with the improved electrical or electrochemical characteristics. Therefore, the introduction of metal nanomaterials can give a large surface area and superior conductivity to enhance the electron transfers for the demonstration of bioelectronic functions. For instance, in one study, the electrochemical signals from a bioelectronic device was remarkably enhanced by the introduction of Au nanoparticles (Au NPs) [33]. In addition, the carbon nanomaterials exhibit different allotropic forms and include various structures such as fullerenes, carbon nanotubes (CNTs), graphene, carbon dots, and nanodiamonds (Fig. 2b) [34]. Because of their inherent flexibility, electrochemical stability, and high carrier mobility, CNTs and graphene have been extensively studied for the development of flexible electronic devices. The lattice structures of CNTs and graphene are particularly useful for increasing mechanical strength and reducing contact resistance [35]. Overall, carbon nanomaterials appear to be promising for developing high-performance and biocompatible electronic devices. TMDs are a family of semiconducting two-dimensional (2D) materials that may potentially make up for the absence of an electronic bandgap in graphene [36]. The chemical formula for TMDs is typically MX_2_, where M stands for a transition metal and X for a chalcogen element such as molybdenum sulfide (MoS_2_) and tungsten sulfide (WS_2_) (Fig. 2c) [37]. TMDs offer a wide range of potential uses in electrochemical biosensors and flexible electronics due to their wide bandgap, atomic-scale thickness, and favorable electrical properties [38]. MXene is a family of 2D transition metal carbides/nitrides and carbonitrides with distinctive mechanical characteristics, high electrical conductivity, and strain-tunable energy storage (Fig. 2d) [39]. MXene has the general chemical formula M_n+1_X_n_T_n_ (n = 1 to 4), where M stands for early transition metals, X for carbon or nitrogen, and T for surface-terminated groups [40]. Because of their excellent hydrophilicity and rich surface chemistry, MXene has a wide range of applications in biomedical engineering. The properties, stability, and functions of MXene could be greatly enhanced by combination with hydrogels [40].

Theoretical and experimental research on the various types of nanomaterials has expanded, and they are now being used in a wide range of fields. However, certain issues remain to be addressed including particle agglomeration or lowering costs for practical applications [41, 42]. Moreover, interactions between the nanomaterials and biomaterials should be managed carefully to maximize the function of the bioelectronic devices.

### Hydrogels

Hydrogels are 3D polymer networks with hydrophilic properties [43]. Hydrogels are widely used in bioelectronics for direct interface with human tissue because of their multifunctionality including biocompatibility, mechanical characteristics, and microenvironment properties [44]. To maximize the functionality of hydrogel bioelectronics, various biological and physical properties should be optimized in accordance with the intended application. Hydrogels can be classified into two categories depending on their origin: natural hydrogels and biocompatible synthetic hydrogels [45]. Natural hydrogels include collagen, gelatin, and Matrigel; these are derived from biological sources and show the highest biocompatibility because they are part of the extracellular matrix (ECM) [46–48]. Biocompatible synthetic hydrogels including poly(acrylamide) (PAAm), polyethylene glycol (PEG), and poly(vinyl alcohol) (PVA) are produced in an artificial way [49–51]. The main advantage of biocompatible synthetic hydrogels is that they are easy to produce with the desired mechanical properties showing high reproducibility because of the straightforward regulation of their synthesis process [52].

**Fig. 3:**
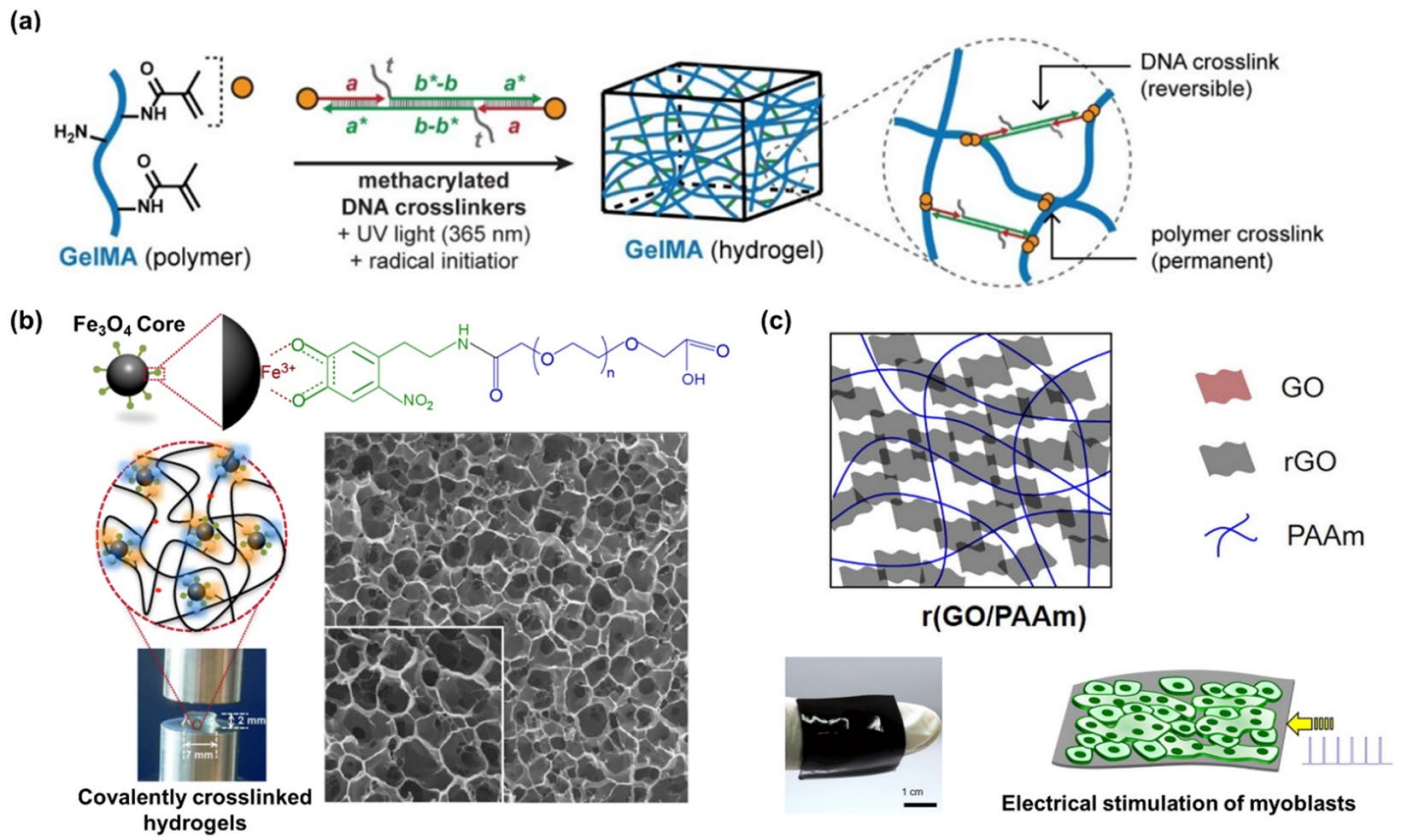
**a** Schematic diagram of the control of hydrogel stiffness by manipulating the crosslinking density using DNA crosslinkers. Reproduced with permission from [56], copyright John Wiley and Sons, 2021. **b** Schematic and optical images of nitro-dopamine-modified MNP incorporated into a collagen-based hydrogel, and SEM image of its internal microstructure. Reproduced with permission from [67], copyright American Chemical Society, 2016. **c** Schematic and optical images of an rGO-based hydrogel nanocomposite and its application for the growth and differentiation of myoblasts. Reproduced with permission from [69], copyright Elsevier, 2016

The properties of hydrogels are ultimately influenced by factors including the preparation methods, degrees of crosslinking, and molecular structures, which are acquired during subsequent processing [53, 54]. Because the properties of the hydrogels used in the development of bioelectronic devices can substantially affect the activity of biomaterials, selecting the appropriate type and properties of the hydrogel for the intended application is critical. Hydrogel stiffness ensures that the hydrogel bioelectronic device is effectively adapted to human tissues [55]. For example, the hydrogels used to develop wearable devices that are connected to the human skin need flexibility, but the hydrogels used to develop implantable devices that are incorporated into bones need considerable stiffness. Hydrogel stiffness is mainly determined by the type of hydrogel, crosslinking density, and gelation conditions. In one study, by incorporation of DNA and hydrogel together, the stiffness of the hydrogel was regulated reversibly by using the displacement of DNA in the hydrogel (Fig. 3a) [56].Here, the DNA performs a role as the crossliner at the inner area of the hydrogel, so the overall stiffness of the hydrogel was successfully regulated via the crosslinking by DNA crosslinker and the displacement of DNA inside the hydrogel. This study showed the possibility of regulation of the hydrogel stiffness reversibly and simply. If the bioelectronic devices are used for the degradation-mediated release of biomaterials or the replacement of organs or tissues, hydrogel biodegradability is required [57]. Most natural hydrogels are naturally degraded by enzymes secreted by the cell. If the hydrogels do not degrade naturally, they can still be broken down by methods such as enzymatic breakdown, hydrolysis, dissolution, and photodegradation [58, 59]. In recent years, several studies have been conducted to incorporate cells into hydrogels and apply them directly to the living body, which requires the careful management of the cell adhesion properties [60]. Cells adjust their behavior including adhesion, morphogenesis, differentiation, and proliferation based on multiple signals from the surrounding cells and microenvironment [61]. Some natural hydrogels can offer a distinct microenvironment for the occurrence of specific cellular behaviors. Furthermore, the porosity of the hydrogel is also crucial for biomaterials to become more active. Highly interconnected porosity in the hydrogel allows for the efficient transport of nutrients, waste products, and gases [62]. In addition, since the presence of pores has a substantial impact on the capacity to absorb water, it is closely related to the mechanical properties of the hydrogel [63].

However, there are some disadvantages of hydrogels during the development of novel bioelectronic devices. For instance, due to the inherent mechanical properties of hydrogels, there is a limitation for the control of mechanical and structural properties of hydrogel-based bioelectronic devices [64]. For this reason, research that aims to overcome these limitations by development of biohybrid hydrogels through the incorporation of nanomaterials into hydrogels has received considerable interest [65, 66]. For example, an extremely low concentration of magnetic nanoparticles (MNPs) modified with nitro-dopamine was incorporated into collagen-based hydrogels to increase their mechanical stiffness without damaging the pore morphology and internal hydrogel structure (Fig. 3b) [67]. Here, the MNPs acted as crosslink epicenters that covalently interacted with the multiple polymeric chains, inducing the formation of a highly crosslinked hydrogel network. Moreover, the mechanical stiffness of the hydrogels was altered within a given range by modifying the size and concentration of the MNPs. Another research examined the effectiveness of Ti_3_C_2_T_x_ MXene in a porous chitosan-hyaluronate hydrogel [68]. Because the Ti_3_C_2_T_x_ MXene decreased the balance between the chemical groups accessible for electrostatic interactions, adding the Ti_3_C_2_T_x_ MXene to the hydrogel dramatically reduced the porosity, which could be advantageous when introducing biomaterials. For instance, when cells are cultured inside a hydrogel, optimizing the porosity of the hydrogel is crucial for the growth and proliferation of the cells. In another study, an electrically conductive hydrogel nanocomposite made of reduced graphene oxide (rGO) and PAAm improved the growth and differentiation of myoblasts by electrical stimulation, facilitated by the adjusted suitable Young’s modulus and electroconductivity of the hydrogel nanocomposite (Fig. 3c) [69]. The properties and applications of the nanomaterials, hydrogels, and biohybrid hydrogels are summarized in Table 1. In addition, research on the development of biohybrid hydrogels of diverse types, shapes, and structures is currently being carried out to enhance the performance of bioelectronic devices [22].

**Table 1:** Application of nanomaterials and hydrogels in bioelectronics

Materials	Components		Properties	Applications	Refs
Nanomaterials	Metal nanomaterials		High biocompatibility, optical polarizability, and catalytic activity	Chemical and biological sensors using surface plasmon resonance (SPR) for real-time and label-free detection of analytes	[31]
	Carbon nanomaterials		Excellent flexibility, electrochemical stability, and high carrier mobility	Microelectrochemical devices, soft sensors, and actuators	[35]
	Transition metal dichalcogenides (TMDs)		Wide bandgap, atomic-scale thickness, and favorable electrical properties	Applications in electronics such as semiconductors and superconductors	[38]
	MXene		Distinctive mechanical strength, high electrical conductivity, and strain-tunable energy storage	Fabrication of flexible devices such as circuit breakers and electrical switches	[40]
Hydrogels	Natural	Collagen	High biocompatibility and biodegradability	Fabrication of 3D scaffolds and biological cues for cell adhesion and proliferation	[46]
		Gelatin	High biocompatibility, biodegradability, non-immunogenicity, and capacity for modification at the amino acid level	Hard- and soft-tissue engineering, drug delivery, and biological glues	[47]
		Matrigel	Providing multiple adhesion sites for cell attachment as well as proteins such as growth factors and transforming growth factors	Culturing of 2D cells and generation and encapsulation of 3D organoids or tissues	[48]
	Biocompatible synthetic	Poly(acrylamide) (PAAm)	High water content, biocompatibility, non-biodegradability, and formation of various shapes	Solid biomedical devices such as contact lenses	[49]
		Polyethylene glycol (PEG)	Superior hydrophilicity, lack of immunogenicity, high biocompatibility, and resistance to protein adsorption	Fabrication of scaffolds for tissue engineering and drug delivery	[50]
		Poly(vinyl alcohol) (PVA)	Excellent hydrophilicity, biodegradability, and biocompatibility	Tissue engineering for regenerating tissues and organs including heart valves, corneal implants, and cartilage tissue substitutes	[51]
Biohybrid hydrogels	Nitro-dopamine-modified magnetic nanoparticle (MNP) with collagen-based hydrogel		Increase in the mechanical stiffness of the collagen-based hydrogel	Controlling single-cellular behavior	[67]
	Ti_3_C_2_T_x_ MXene-incorporated chitosan-hyaluronate hydrogel		Control of the porosity of the chitosan-hyaluronate hydrogel	Imparting antibacterial properties to the hydrogel	[68]
	Reduced graphene oxide (rGO)-incorporated PAAm hydrogel		Increase in the electrical conductivity of the PAAm hydrogel	Simultaneous delivery of electrical and mechanical cues to biological systems	[69]

### Biohybrid hydrogel for flexible/wearable bioelectronic devices

**Fig. 4:**
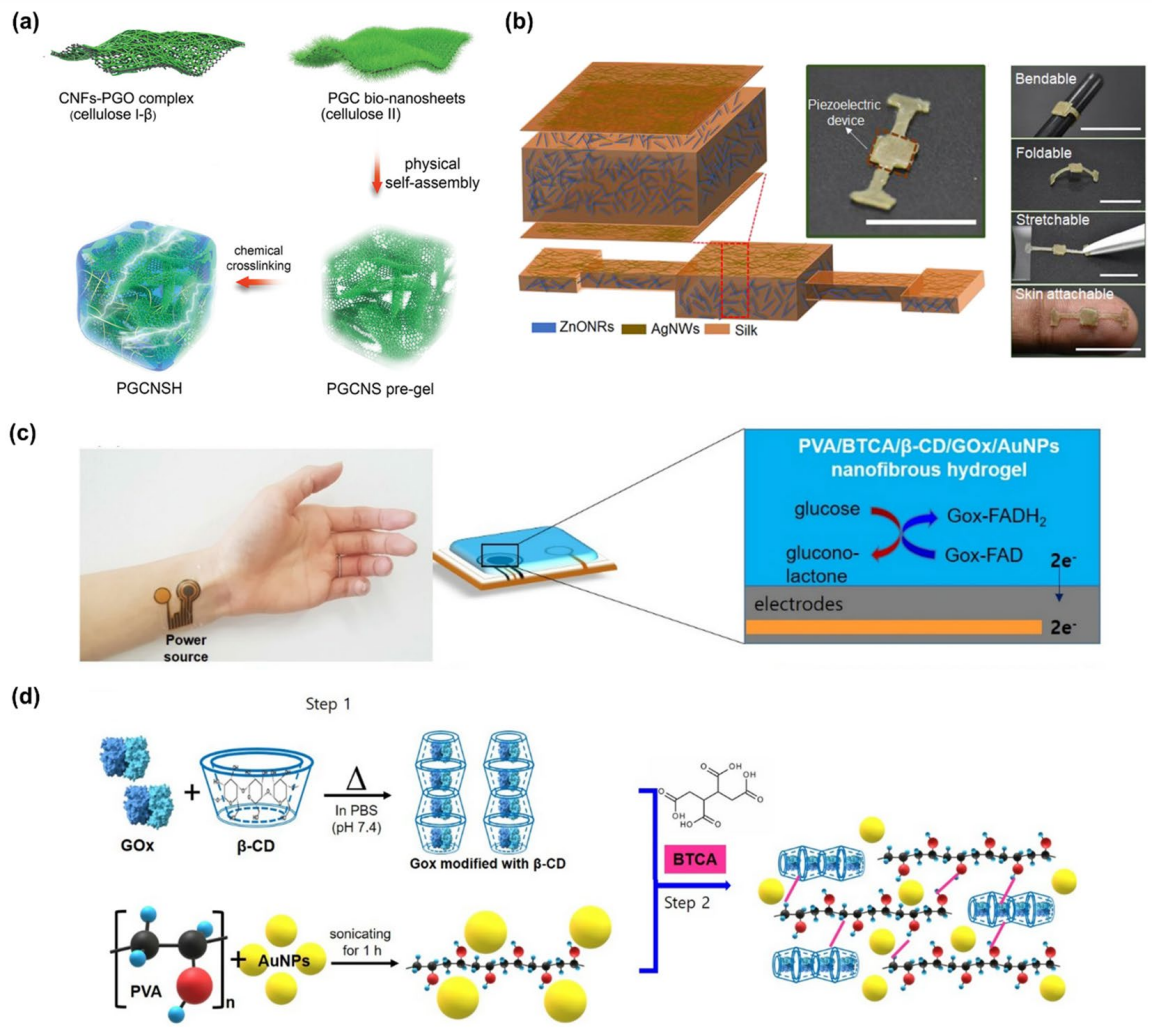
**a** Schematic image for the development process of pure regenerated cellulose-based hydrogel (PGC bionanosheet-assembled hydrogel (PGCNSH)) via chemical crosslinking of a (PGO)-hybridized cellulose bionanosheet (PGCNS) by using ECH. Reproduced with permission from [70], copyright John Wiley and Sons, 2021. **b** Scheme and optical images of artificial electronic skin composed of engineered silk protein hydrogel, ZnO NRs, and Ag NWs, and its flexibility and adhesion properties. Reproduced with permission from [77], copyright Elsevier, 2020. **c** Optical image and biosensing mechanism of a wearable electrochemical glucose biosensor composed of a transparent nanofiber-type hydrogel with encapsulated Au NPs and GOx, and **d** Schematic illustration of the steps for developing a transparent nanofiber-type hydrogel with encapsulated Au NPs and GOx for use in electrospinning. Reproduced with permission from [83], copyright Springer Nature, 2020

Research on hydrogel bioelectronics has focused on the efficient conjugation of nanomaterials and hydrogels to develop the biohybrid hydrogels. The conductive and biocompatible biohybrid hydrogels have been applied in the development of functional bioelectronic devices, biofuel cells, and electrochemical and electrical biosensors. Various nanomaterials including those based on carbon and metal have been applied in the development of biohybrid hydrogel bioelectronics [22]. For example, a polydopamine-rGO (PGO)-hybridized cellulose bionanosheet (PGCNS) was combined with hydrogel for producing a bioelectrode (Fig. 4a) [70]. The presynthesized PGO-assisted cellulose nanofibrils (PGC) were combined with PGO, and the prepared bionanosheet was chemically crosslinked using epichlorohydrin (ECH) to form a pure regenerated cellulose-based hydrogel (PGC bionanosheet-assembled hydrogel (PGCNSH)). In another example, a mixture composed of catechol lignin (DAL) and graphene oxide was conjugated with a sodium alginate/polyacrylamide (SA/PAM) hydrogel. The developed conductive hydrogel exhibited excellent adhesion to the human skin, electrical conductivity, and bendability [71]. In addition, a biomimetic α-helical peptide hydrogel (a single-type biomolecular hydrogel) was developed and its electrical conductivity was investigated [72]. By combining with nanomaterials to produce nanobiohybrid materials, the applicability of natural hydrogel in bioelectronics will be broadened. In particular, the flexible properties of hydrogel greatly increase its utilization in the development of wearable or flexible bioelectronic devices [73].

Researchers imparted field-effect transistor (FET) characteristics to a device using hydrogel, which directly conferred conventional electronic functions [74]. In one study, a bulk DNA hydrogel was developed and utilized as a solid-state electrolyte on an Au electrode to provide organic FET functions, and a DNA aptamer capable of targeting a vascular endothelial growth factor (VEGF) was introduced in the bulk DNA hydrogel-based FET to detect the VEGF in a FET manner. In another study, bioreceptors (penicillinase and acetylcholinesterase) were encapsulated in the hydrogel, which was used as a gate for the FET to demonstrate multiplexed biosensing at a nanoscale [75]. Moreover, electrical skins were developed using a conductive cellulose liquid–crystal hydrogel composed of CNT, hydroxypropyl cellulose, and poly(acrylamide-co-acrylic acid) [76]. The developed hydrogel complex exhibited unique optical properties and responded to external stimuli including temperature changes, applied pressure, and tension in a colorimetric way as an electrical skin. Likewise, a self-powered artificial skin platform based on an engineered silk protein hydrogel was reported (Fig. 4b) [77]. To develop the artificial electronic skin, the silk protein was engineered to produce a hydrogel, and it was then combined with zinc oxide nanorods (ZnO NRs) and Ag nanowires (Ag NWs) to develop an artificial piezoelectric energy-generating electronic skin. The developed electronic skin showed outstanding flexibility and piezoelectric response with high efficiency. Furthermore, the skin produced electrical energy with high power density by motion (1 mW/cm^2^). In addition to these studies, in recent years, hydrogel has been widely used in the field of bioelectronics and its potential is limitless. For instance, a textile electrode composed of a polymer hydrogel electrolyte and carbon nanomaterial was developed for manufacturing a flexible enzymatic biofuel cell [78].

In addition, research on the development of biohybrid hydrogel bioelectronic devices has resulted in the development of various electrochemical and electrical biosensors [79]. Research on wearable or flexible biosensors, particularly those made of hydrogel, is attracting increased attention because of the outstanding flexibility of the latter. Through the incorporation of nanomaterials and hydrogel, a synergistic effect can be obtained in the development of flexible or wearable biosensors by enhancing conductivity and bendability. Moreover, by combining biomolecules and functional hydrogel, highly sensitive electrochemical biosensors can be developed. For instance, an electrochemical biosensor composed of hydroxyethylmethacrylate (HEMA) and 2-(diethylamino)ethyl methacrylate (DEAEMA) hydrogels and enzymes (glucose oxidase (GOx) and acetylcholinesterase) was incorporated to Au screen-printed electrodes (SPE), using a hydrogel-coating technique based on plasma polymerization [80]. Another study showed the possibility of using hydrogel as an electrode for electrochemical biosensors by developing an effective 3D electron transporter composed of DNA hydrogel [81]. In addition, inspired by human muscles, a self-healing binary-network hydrogel composed of polyacrylic acid (PAA)-polyaniline (PANI) nanofibers and metal ions was developed for the demonstration of strain and temperature biosensing [82]. A group of researchers developed a wearable electrochemical glucose biosensor using transparent nanofiber-type hydrogel with encapsulated Au NPs and GOx (Fig. 4c) [83]. In this study, the base polymers PVA and β-cyclodextrin (β-CD) was used. The cyclic oligomer β-CD has cavities in which GOx could be inserted, stabilizing the activity of the latter for efficient glucose detection. 1,2,3,4-Butanetetracarboxylic acid (BTCA) was introduced as a crosslinking agent for hydrogel formation, and Au NPs were incorporated to enhance the electrochemical sensitivity of the GOx-encapsulated hydrogel (Fig. 4d). Then, using the electrospinning method, a transparent nanofiber-type hydrogel was developed, and this wearable electrochemical glucose biosensor exhibited biocompatibility, flexibility, and glucose detection capability. In addition, research on hydrogels for flexible or wearable bioelectronic applications is currently being conducted for practical application. For example, an ultraconductive filler-free polymeric hydrogel was developed [84] and is expected to serve as a foundation for the commercialization of flexible or wearable hydrogel bioelectronic devices.

### Biohybrid hydrogel for tissue engineering (3D organ or 2D cell scaffold)

Typically, soft and porous hydrogels with a high water content are capable of mimicking ECM and offer a comparable microenvironment for cell growth, nutrition delivery, and formation of biological tissue [85, 86]. To enhance properties like cell adhesion, electrical conductivity, and stimuli responsiveness, hydrogels have been incorporated with nanomaterials and utilized for direct implantation into biological tissues [87].

**Fig. 5:**
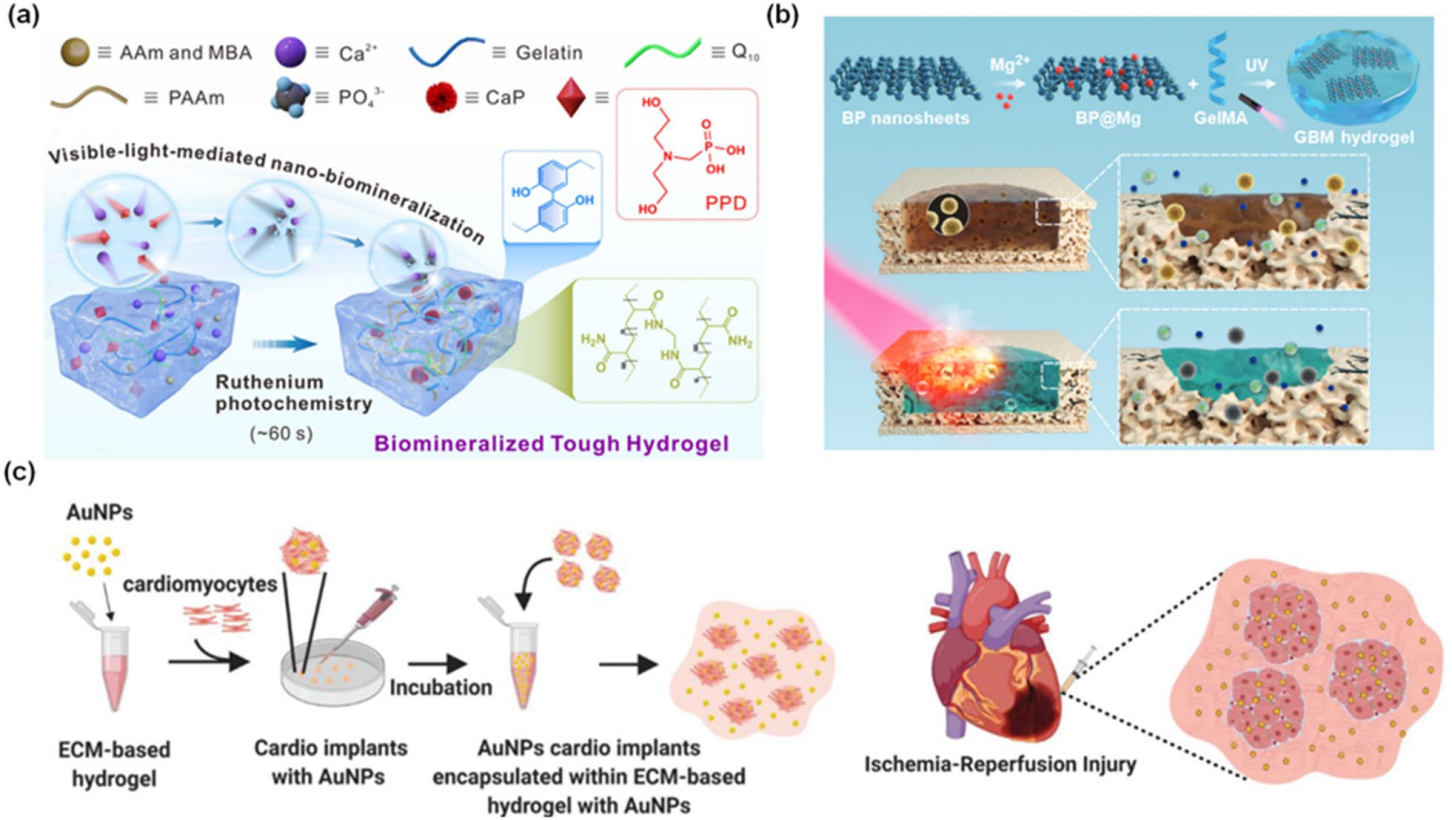
**a** Schematic diagram of a visible-light-mediated nano-biomineralization method for the synthesis of BTH through the photocatalytic activity of ruthenium. Reproduced with permission from [88], copyright American Chemical Society, 2022. **b** Schematic images of the fabrication process of GelMA-BP@Mg composed of GelMA and BP@Mg and exhibiting the photothermal properties of the GelMA-BP@Mg. Reproduced with permission from [90], copyright John Wiley and Sons, 2021. **c** Schematic images of the fabrication process of ECM-Au NP-based composite hydrogel and encapsulation of cardiac cells for heart disease (ischemia–reperfusion) treatment. Reproduced with permission from [94], copyright John Wiley and Sons, 2021

With this approach, a visible-light-mediated nano-biomineralization method for the one-step synthesis of customizable biomineralized tough hydrogel (BTH) was reported (Fig. 5a) [88]. Acrylamide (AAm) and gelatin generated a multinetwork-structured tough hydrogel (TH), and simultaneously, upon exposure to blue light (452 nm), phosphate ions (PO_4_^3−^) were precipitated by Ca^2+^ metal ions in conjunction with photocatalysis using ruthenium, forming calcium phosphate nanoparticles (CaP NPs). The TH was nano-mineralized during the formation process of the CaP NPs, and the BTH was synthesized in as little as 60 s under visible light. Also, the BTH precursors contained the water-soluble phosphonodiol (PPD), so after synthesis, the BTH included the PPD with water-solubility. Furthermore, due to mineralization, the BTH exhibited enhanced physical properties such as maximum stress of 0.5 MPa together with a critical strain of 800% and toughness of 2.0 MJ/m^3^ to make up the defects caused by damaging of biological tissue. The customized BTH was implanted into damaged skin and skull defects as a proof-of-concept demonstration. The implanted BTH alleviated inflammation in the surrounding tissues and promoted the cell adhesion, proliferation, and differentiation necessary for tissue repair. Beyond simply supporting tissue regeneration by introducing hydrogels, more recent work has focused on providing bioelectronic functionality by implanting biohybrid hydrogels [89]. In one study, the gelatin methacryloyl (GelMA) was combined with magnesium-modified black phosphorus nanosheets (BP@Mg) to fabricate a photosensitive conductive GelMA-BP@Mg (GBM) hydrogel for healing infected bone (Fig. 5b) [90]. When exposed to NIR light, the GBM hydrogel displayed effective antibacterial effects and decreased the inflammation caused by infection because of the photothermal properties of the BP@Mg. These results indicated the formation of a favorable microenvironment for the regeneration of infected bone by eliminating bacteria. Using conductive materials to stimulate innervated bone regeneration is a promising approach because the electrical impulses produced by the endogenous electrical fields provide benefits for tissue regeneration [91]. Due to the addition of BP@Mg, the GelMA-BP@Mg was more conductive than the GelMA hydrogel. The GelMA-BP@Mg acted as a conductor and exhibited excellent conductivity that was enough to turn on a blue light-emitting diode (LED). The efficiency of bone regeneration was substantially increased by triggering the neural network reconfiguration and bone differentiation in the injured bone section, owing to the synergistic antibacterial and high conductivity effects.

In another study, enhancement of the electrical conductivity of a biohybrid hydrogel was achieved for the implementation of the biohybrid hydrogel as a nerve signal sensor [92]. Here, an ultrafast and biocompatible digital patterning method was developed through phase separation induced by a laser for developing a poly(3,4-ethylenedioxythiophene):poly(styrene sulfonate) (PEDOT:PSS) hydrogel. The laser was irradiated on the PEDOT:PSS hydrogel composed of a PSS-shell and PEDOT-core to separate the phase into two regions: a connected-PEDOT-rich domain and a PSS-rich domain. Moreover, to increase the interaction between the PEDOT:PSS hydrogels and the laser, Au NPs were introduced. The extended and connected PEDOT-rich domains formed by phase separation substantially improved the electrical conductivity and water stability of the PEDOT:PSS hydrogel. This allowed the transformation of the PEDOT:PSS hydrogels into water-stable hydrogels. Furthermore, in a physiological environment, the electrochemical properties were maintained even after six months. Using the developed PEDOT:PSS hydrogel, stable neural signal recording and stimulation were demonstrated. Likewise, another study reported the development of an MXene/regenerated silk fibroin (RSF) nanocomposite hydrogel [93]. The MXene/RSF nanocomposite hydrogel was fabricated by adding 2D bioencapsulated MXene nanosheets into an RSF solution to form a dual-crosslinked network, using horseradish peroxidase (HRP) and hydrogen peroxide (H_2_O_2_). The multiple tunable functional groups on the surface of MXene provided the properties of high water dispersion, plasticity, and the ability to construct different composites and microstructures by fusing with other materials. The RSF nanofibrils were induced to self-assemble on the MXene nanosheets as a nanostructured template. When the MXene concentration reached 8 mg/mL in the MXene/RSF nanocomposite hydrogel, the electric conductivity increased to around 4 × 10^–4^ S/cm, demonstrating the electroactivity of the developed hydrogel. A cut-contact test was also conducted to investigate the conductivity of the MXene/RSF nanocomposite hydrogel. The resistance of the MXene/RSF nanocomposite hydrogel quickly increased to a stable open-circuit condition when the hydrogel circuit was cut off. However, the original value was recovered after the two separated hydrogels were recontacted, demonstrating the uniform distribution of MXene throughout the RSF hydrogel. Using the developed MXene/RSF nanocomposite hydrogel, the mechanism by which electrical stimulation promotes osteogenic differentiation was also explored. Using a critical-size calvarial bone lesion model, the therapeutic potential of the hydrogel for bone regeneration and neovascularization was examined in vivo. Although the regeneration of bone defects is the primary application of tissue engineering using biohybrid hydrogels, reports of the introduction of biohybrid hydrogels into other tissues can be found in the recent literature. For example, an ECM-Au NP-based composite hydrogel capable of aiding heart regeneration was developed (Fig. 5c) [94]. The ECM-based natural hydrogel was prepared by the decellularization of omenta from healthy pigs and the incorporation of Au NPs to fabricate a scaffolding material with fast conduction velocity. This material enabled the fast transfer of electrical signals between the adjacent cardiac cells. Moreover, a low concentration of cardiac cells was encapsulated into the prepared composite hydrogel, forming an injectable tissue engineering system. After being injected into a mouse model of ischemia–reperfusion, the developed cardiac cell-containing composite hydrogel decreased the scar size and prevented the deterioration of the heart function.

Generally speaking, it is very difficult to develop hydrogels with tissue-dependent mechanical strength, cell adhesion and signaling, and suitable biodegradability for application in tissue engineering [95]. Thus, developing biohybrid hydrogels that combine the advantageous traits of the hydrogels with those of complementary nanomaterials is advantageous. As discussed in this section, the unique properties of nanomaterials such as their photothermal effect and electrical conductivity, not only compensate for the flaws of the hydrogels but also directly affect tissue regeneration and signal recording. Recently, the outstanding potential of 3D printing technology to produce complex structures with high accuracy has attracted a lot of attention, particularly in tissue engineering for skeletal, maxillofacial, and otorhinolaryngology applications [96–99]. For example, a study reported the development of GelMA/laponite-based hydrogel scaffolds using 3D-printing techniques [100]. Scaffolds with appropriate structures can be made easily using 3D printing, owing to the simplicity of the customized manufacturing process. In particular, major developments in 3D-printing technology, combined with the incorporation of biohybrid hydrogel for patient-specific regeneration treatments, are expected to have a notable impact on the rapidly developing field of personalized medicine.

### Biohybrid hydrogel for biorobotics

**Fig. 6:**
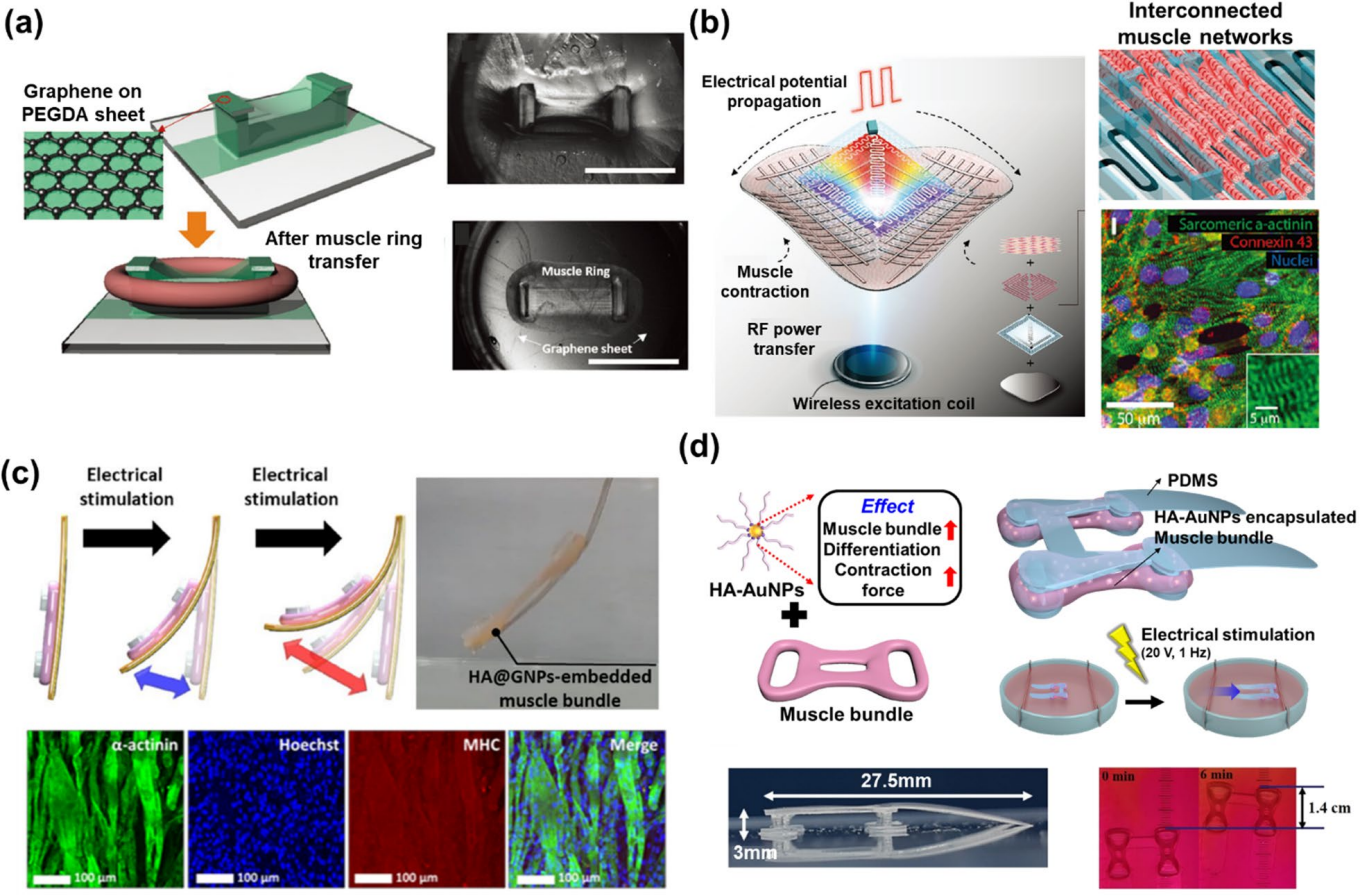
**a** Schematic diagram of a conductive hydrogel electrode with a 3D tissue model for the application of electrical stimulation to induce muscle contraction. Reproduced with permission from [104], copyright John Wiley and Sons, 2020. **b** Schematic diagram of a wirelessly powered 3D-printed hierarchical biohybrid robot composed of four parts: (i) A CNT/GelMa hydrogel layer for the elastic body, (ii) stretchable wirelessly-powered bioelectronics for cell stimulation, (iii) 3D-printed accordion-inspired scaffolds, and (iv) iPSC-derived muscle tissues. Reproduced with permission from [106], copyright John Wiley and Sons, 2022. **c** Schematic diagram of the actuation process of an electroactive nanobiohybrid actuator composed of MoS_2_ nanosheets and a hydrogel-based skeletal muscle structure. Reproduced with permission from [108], copyright Springer Nature, 2022. **d** Schematic diagram of a biohybrid robot composed of HA-Au NPs-embedded muscle hydrogel. Reproduced with permission from [110], copyright American Chemical Society, 2022

Biorobotics is one of the research areas where hydrogel bioelectronics can be applied, for instance, in biorobot-based drug screening and disease model demonstration. Moreover, the inherent properties of hydrogels, such as their biocompatibility and porous structure, create suitable environments for the myogenic differentiation of muscle cells into myotubes, which is required for the development of biorobots because they are normally operated by muscle contraction [101]. Due to these properties, biohybrid hydrogel-based muscle structures have shown high contraction performance compared to conventional 2D muscle cell sheets. Biorobots can be driven by controlling the movement of the biohybrid hydrogel-based muscle structure by electrical stimulation or by transmitting electrophysiological signals to induce muscle contraction. For example, an antagonistic pair of skeletal muscle tissues were produced for operating a biorobot [102]. The skeletal muscle tissue was prepared using polydimethylsiloxane (PDMS) stamps and immobilized on a plastic plate, which was employed as an anchor to fabricate the biohybrid robot. To fabricate the skeletal muscle tissue, myoblasts were mixed with Matrigel to form a myoblast-laden hydrogel sheet. The developed biohybrid robot was controlled by applying electrical stimulation with Au electrodes. In another example, tethering myoblasts with rGO were combined with Matrigel and fibrinogen to form a 3D myoblast ring for a biohybrid pump, which increased the fluid velocity and flow rate in a similar way as conventional pump equipment [103]. The fabricated rGO-tethered myoblast exhibited superior muscle contraction as a result of the electrical conductivity of rGO. In addition, the integration of a conductive hydrogel electrode with a 3D tissue model was reported to result in electrical stimulation for inducing muscle contraction [104]. The graphene was transferred to a rigid poly(ethylene glycol) diacrylate (PEGDA) layer by chemical vapor deposition for developing a conductive hydrogel electrode. Due to the introduction of the graphene, it granted the conductivity to the PEGDA for developing the conductive hydrogel electrode, so the effective electrical stimulation to muscle cells was demonstrated by this electrode well. The conductive hydrogel electrode was integrated with the 3D skeletal muscle ring, which was a ring-shaped structural muscle bundle composed of C2C12 skeletal muscle cells and Matrigel. Because the 3D skeletal muscle ring can be controlled by electrical stimulation with various applied voltages and frequencies, it was actuated electrically through the conductive hydrogel electrode (Fig. 6a).

In addition, many studies have been performed to develop actual biorobots, rather than to partially apply a hydrogel-based muscle structure. In one study, a walking biohybrid robot was developed using a 3D-printed PEGDA hydrogel skeleton [105]. The walking biohybrid robot was prepared using hydrogel scaffolds and muscle tissue. Because of the flexibility of the 3D-printed PEGDA hydrogel skeleton, the muscle tissue composed of C2C12 skeletal muscle cells and ECM was easily actuated with electrical stimulation. A paper reported a wirelessly powered 3D-printed hierarchical biohybrid robot [106]. The hierarchical biohybrid robot was composed of four parts: (i) A CNT/GelMa hydrogel layer for the elastic body, (ii) stretchable wirelessly powered bioelectronics for cell stimulation, iii) 3D-printed accordion-inspired scaffolds, and iv) induced pluripotent stem cell (iPSC)-derived muscle tissues. Due to the direct employment of stretchable wirelessly powered bioelectronics, the iPSC-derived muscle was controlled by electrical stimulation without requiring batteries or wires (Fig. 6b). The developed biohybrid robot indicated a maximum locomotion speed of 580 µm/s and electrically controlled the swimming motion of the muscle tissues. This research demonstrated the advantage of using biohybrid hydrogel in the operation of biorobots. In another example, a bioinspired soft robot was developed using CNT/GelMA hydrogel and cardiomyocytes [107]. To develop this bioinspired soft robot, the CNT/GelMA hydrogel was 3D printed on the PEG hydrogel with an Au electrode for inducing the contraction of cardiomyocytes by electrical stimulation. To achieve the cell alignment so that the cell–cell interaction and beating efficiency of the cardiomyocytes would be enhanced, CNT/GelMA and PEG hydrogel patterns with a spacing of 75 and 300 µm, respectively, were introduced. Furthermore, this bioinspired soft robot was electrically actuated by applying voltage and showed a strong bending behavior over 7 days.

Among the studies on biorobots, some reports have described the introduction of nanomaterials directly into the muscle cell structure for enhancing the contraction of the muscle cells so as to achieve sufficient energy for driving the biorobots. For example, an electroactive nanobiohybrid actuator composed of MoS_2_ nanosheets and a hydrogel-based skeletal muscle structure was developed to enhance the driving motion of a biorobot [108]. For this purpose, the MoS_2_ nanosheet-based electrochemical actuator was introduced to the hydrogel-based skeletal muscle structure using a PDMS support pillar. The motion performance of the nanobiohybrid actuator was generated by combining the contraction of the hydrogel-based skeletal muscle structure and the movement of the MoS_2_ nanosheet-based electrochemical actuator. The developed electroactive nanobiohybrid actuator exhibited 3.18 times higher motion performance than that of the hydrogel-based skeletal muscle structure alone, which demonstrated the beneficial effect of incorporating nanomaterials into hydrogels for the development of biorobots (Fig. 6c). In another example, a cardiac tissue engineered-bioactuator was developed using rGO-GelMA hydrogel and cardiomyocytes [109]. The rGO-GelMA showed a highly porous microstructure compared to the pristine GelMA hydrogel, which substantially enhanced the spontaneous beating rate of the cardiomyocytes. In addition to the above-described biorobots with improved motion performance, biorobots can also be used in biomedical applications, for example, to demonstrate muscle-related diseases or to monitor muscle responses to external chemical stimuli such as drug treatments, and can thus provide a platform for drug efficiency tests or disease models. For instance, a biohybrid robot composed of hyaluronic acid-modified Au NPs (HA-Au NPs)-embedded muscle hydrogel was used for investigating the effects of an inotropic drug [110]. Here, the Au NPs contributed to improve the conductivity of the hydrogel for achieving the effective actuation by electrical stimulation. Also, the HA contributed to the efficient growth and differentiation of muscle cells. Therefore, by incorporation of HA and Au NPs, the synergic effects of HA and Au NPs in the HA-Au NPs-embedded muscle hydrogel enhanced the muscle cell proliferation and differentiation due to the high water retention capacity and hydration ratio of the hydrogel composite, and enhanced the movement of the developed biohybrid robot by electrical stimulation (Fig. 6d). By investigating the movement of the biohybrid robot based on the electrical stimulation, the effect of the inotropic drug (a drug used for controlling muscle contraction) was investigated. To monitor the positive and negative effects of the inotropic drug, 100 nM isoprenaline and 10 µM verapamil were added to the biohybrid robot. The velocity of the biohybrid robot increased 1.5 times by the positive effect of isoprenaline and decreased 5.73 times by the negative effect of verapamil. As seen in this section, numerous studies have been conducted to develop hydrogel-based biorobots or actuators, using various nanomaterials and muscle cells. However, since hydrogel-based biorobotic research is in its infancy, many questions remain to be addressed. For instance, hydrogels with ultralight weight but high Young’s modulus and conductivity are required to develop ultralight-weight biorobots able to move effectively. Thus, we expect that research on novel hydrogels with enhanced properties will be conducted along with the development of biorobots.

## Conclusion

Hydrogel bioelectronics are receiving increased attention because they allow electronics to function in a highly biocompatible manner [44]. Although numerous types of hydrogels are being actively researched for diverse applications in bioelectronics, the intrinsic limitations of hydrogels including their unstable structural properties, low conductivity, and poor cell adhesion hinder the development of novel bioelectronic devices [64]. Therefore, various types of nanomaterials are being combined with hydrogels to overcome these problems, and the biohybrid hydrogels exhibit synergic effects as a result of the incorporation of the unique properties of nanomaterials into hydrogels [111].

This review summarized recent research on biohybrid hydrogels in bioelectronics. First, the structures and characteristics of metal nanomaterials, carbon nanomaterials, TMDs, and MXene were discussed. Next, the different types of hydrogels and their required properties for the development of bioelectronics were discussed, and the advantages of the incorporation of nanomaterials into hydrogels were provided. Afterward, the bioelectronic applications of biohybrid hydrogels were discussed by following the categorized sections as flexible/wearable bioelectronic devices, tissue engineering, and biorobotics. The bioelectronic devices based on biohybrid hydrogel discussed in this review are summarized in Table 2. In addition to the subjects discussed here, biohybrid hydrogels are being actively researched for other bioelectronic applications. For example, self-healing hydrogels composed of glycol chitosan and GO showed improved self-healing function (more than 99% recovery) and excellent mechanical properties compared to conventional pristine hydrogels [112]. Another study reported the photo-directed morphing structure of shape memory hydrogels that can transform into various configurations by photo-triggered site-specific deformations and can be used to fabricate programmable hydrogel actuators [113]. Despite these recent applications, challenges still remain to be addressed before biohybrid hydrogels can be used for manufacturing bioelectronic devices. For example, toxicity or harmful effects may occur in the long run when the bioelectronic devices are directly attached to an organ or implanted into the human body [114]. Thus, it is necessary to further enhance the biocompatibility of biohybrid hydrogels to ensure they can be used in harmony with the physiological environment. For instance, the encapsulation of bioelectronic devices with more biocompatible and non-toxic materials or the chemical modification of surface of bioelectronic devices with biocompatible chemical molecules may be helpful to reduce the possibility of toxic or side effects of the bioelectronic devices duing the implantation of them in living things. Also, in order to incorporate the nanomaterials and hydrogels efficiently, the appropriate surface modification of each component with proper chemical groups may enhance the interaction between them to develop the novel biohybrid hydrogels to be applied in bioelectronics. Moreover, because the bioelectronic devices composed of biohybrid hydrogels show variations in their electrical and biological stability, a strategy should be devised for preserving their stability in diverse environments [115]. We also expect that future biohybrid hydrogels will be applied in various scientific disciplines beyond bioelectronic applications. In conclusion, this review highlights the recent trends in biohybrid hydrogels and their applications in bioelectronics. These achievements are anticipated to be a milestone and provide an innovative and insightful approach toward the development of next-generation bioelectronic devices and biohybrid robot systems.

**Table 2:** Bioelectronics using biohybrid hydrogels

Application of biohybrid hydrogel in bioelectronics	Hydrogel components	Types of biomaterials	Types of nanomaterials	Role of nanomaterials	Refs
Biohybrid hydrogel in flexible/wearable devices	PGC bionanosheet-assembled hydrogel (PGCNSH)	Cellulose nanofibrils	Polydopamine-reduced-graphene oxide (PGO)	Conductivity enhancement	[70]
	Engineered silk protein hydrogel	Silk protein	Zinc oxide nanorods (ZnO NRs) and Ag nanowires (Ag NWs)	Conductivity enhancement	[77]
	Poly(vinyl alcohol) (PVA), β-cyclodextrin (β-CD), and 1,2,3,4-butanetetracarboxylic acid (BTCA)	Glucose oxidase (GOx)	Au nanoparticles (Au NPs)	Conductivity enhancement	[83]
Bioybrid hydrogel in tissue engineering	Acrylamide (AAm) and gelatin	-	Calcium phosphate nanoparticles (CaP NPs)	Physical properties enhancement	[88]
	Gelatin methacryloyl (GelMA)	-	Magnesium-modified black phosphorus nanosheet (BP@Mg)	Impart photothermal properties and increase conductivity	[90]
	Extracellular matrix (ECM)-based natural hydrogel prepared by decellularization of omenta	Cardiac muscle cells	Au nanoparticles (Au NPs)	Promotion of fast transfer of electrical signals between cardiac cells	[94]
Biohybrid hydrogel in biorobotics	Poly(ethylene glycol) diacrylate (PEGDA) and Matrigel	Skeletal muscle cells	Graphene	Conductivity enhancement	[104]
	Gelatin methacryloyl (GelMA) and polyethylene glycol (PEG)	iPSC-derived muscle tissue	Carbon nanotubes (CNTs)	Conductivity enhancement	[106]
	Matrigel	Skeletal muscle cells	Molybdenum disulfide nanosheets (MoS_2_ NSs)	Conductivity enhancement	[108]
	Matrigel	Skeletal muscle cells	Au nanoparticles (Au NPs)	Cell proliferation and differentiation	[110]

## Data Availability

Not applicable.
